# The effects of high lumbar epidural analgesia on postoperative pulmonary function tests in liver transplant donor patients

**DOI:** 10.1186/2197-425X-3-S1-A693

**Published:** 2015-10-01

**Authors:** HK Atalan, B Gucyetmez, R Donmez, M Berktas, A Kargi, A Erturer, İR Sozenoglu, TB Denizalti, KY Polat

**Affiliations:** Atasehir Memorial Hospital, Anesthesiology and Reanimation, Istanbul, Turkey; Acibadem International HospitaI, Anesthesiology and Reanimation, Istanbul, Turkey; Transplantation Department, Atasehir Memorial Hospital, Istanbul, Turkey; PEPIRC, Yeditepe University, Istanbul, Turkey

## Introduction

Epidural analgesia has positive effects on pulmonary function tests (PFT). ([1]) Besides, it decreases anesthesia requirement, myocardium oxygen consumption, relapsing time of bowel movements, thrombosis risk and blood loss.([2, 5])

## Objectives

The objective of the study was to compare HLEA and general anesthesia combination vs general anesthesia on postoperative PFT in the liver transplant donor patients.

## Methods

Upon the approval of local ethical committee, this prospective randomised study was performed between October 2014 and April 2015. Liver transplant donor patients with ASA I-II status between age of 18-60 years were included. Patients in Group 1 (G1) received total intravenous anesthesia with propofol and remifentanil infusion (10 mg/kg/h and 0,1-0,25 mcg/kg/min) and those in Group 2 (G2) received propofol and HLEA (10 mgr/kg/h and 7 ml/h epidural infusion-*bupivacaine 0,25% plus fentanyl 4*mg/ml in saline). G1 received tramadol (0,15 mg/kg/h and 0,2 mg/kg IV bolus) and G2 received HLEA (5 ml/h) for postoperative analgesia. Patient's age, gender, height, weight, APACHE II scores, induction and maintenance dose of propofol (IDP and MDP), length of time for the surgery, preoperative and postoperative 24^th^ hour FEV_1_, FVC, FEV_1_/FVC ratio, visual pain scores (VPS) at ICU admission, 2^nd^, 4^th^, 8^th^, 12^th^, 18^th^ and 24^th^ hours, length of ICU stay (LOS ICU) and length of hospital stay (LOS H) were recorded. Delta values for PFT were calculated. Groups were compared by using Mann Whitney U test due to non-normal distribution pattern.

## Results

Groups (33 patients in each group) were similar in terms of demographic data, surgery duration, APACHE II scores, preoperative FEV1, FVC and FEV1/FVC ratio and LOS ICU. Median IDP (1.8 mg/kg/h vs 2.2 mg/kg/h), MDP (4.9 mg/kg/h vs. 5.8 mg/kg/h), delta-FEV_1_ (-1.2% vs. -6.8%), delta-FVC (-1.8% vs. -7.4%), LOS Hospital (7.0 days vs 8.0 days) in G2 was significantly lower than G1 (p < 0.001 for each). Moreover median VPS of G2 at beginning, 2^nd^, 4^th^, 8^th^, 12^th^, 18^th^ and 24^th^ hour of ICU admission was also significantly lower than G1 (p < 0.001 for each time point).

## Conclusions

Using HLEA in liver transplant donor patients provided better preservation of FEV_1_ and FVC by decreasing perioperative anesthesia requirement and providing effective postoperative analgesia. Moreover it seemed to decrease length of hospital stay, hence reduce transplantation costs. In this patient group, HLEA can be preferred since it is easy to perform, decreases anesthesia requirement and has beneficial effects on PFT compared to intravenous analgesia, by providing effective postoperative analgesia.
